# A Two-Year Pharmacovigilance Analysis of Adverse Drug Reactions Reported from a University Allergy Setting

**DOI:** 10.3390/jcm15020848

**Published:** 2026-01-20

**Authors:** Paola Maria Cutroneo, Ilaria Marando, Stefania Isola, Angela Alibrandi, Marco Casciaro, Paola Lucia Minciullo, Edoardo Spina, Sebastiano Gangemi, Luisa Ricciardi

**Affiliations:** 1 Unit of Clinical Pharmacology, G. Martino University Hospital of Messina, 98125 Messina, Italy; ilaria.marando@unime.it; 2 Allergy and Clinical Immunology Unit of G. Martino Hospital, Department of Clinical and Experimental Medicine, University of Messina, 98122 Messina, Italy; stefania.isola@unime.it (S.I.); marco.casciaro@unime.it (M.C.); paolalucia.minciullo@unime.it (P.L.M.); gangemis@unime.it (S.G.); luisa.ricciardi@unime.it (L.R.); 3 Department of Human Pathology in Adult and Developmental Age “Gaetano Barresi”, University of Messina, 98122 Messina, Italy; angela.alibrandi@unime.it; 4 Department of Clinical and Experimental Medicine, University of Messina, 98122 Messina, Italy; edoardo.spina@unime.it

**Keywords:** pharmacovigilance, adverse drug reaction, antibiotics, anti-inflammatory drugs, allergy, immediate reaction, delayed reaction

## Abstract

**Background:** Adverse Drug Reactions (ADRs) are a significant public concern because of their impact on healthcare systems. Spontaneous reporting of ADRs is crucial for monitoring drug safety and recognizing possible risk factors. The objective of this study was to characterize ADR reports from the Allergy and Clinical Immunology Unit of the G. Martino University Hospital, Messina, Italy. **Methods:** A retrospective analysis was conducted, including all ADRs spontaneously reported from patients attending the clinic because of at least one previous ADR, from June 2022 to June 2024. **Results**: A total of 388 reports were collected, mainly from females (71.1%) and adult patients (84.3%). ADRs were mostly immediate, from antibiotics and anti-inflammatory drugs (61.5%), with a high prevalence of cutaneous and respiratory disorders. Delayed reactions were mostly from endocrine therapies, vaccines, and antiepileptics. Anaphylactic shock was present only in 13 ADR reports (3.35%). A higher risk of developing serious ADRs was found in elderly patients aged ≥65 years (*p* = 0.012). An original finding was that a positive history of allergies (*p* = 0.023) and past medical history of ADRs (*p* = 0.045) were negatively correlated to the occurrence of a serious ADR, probably because patients had been previously followed in an allergy setting and alerted about ADRs. **Conclusions:** This study underlines the role of ADR follow-up in allergy settings to identify preventable traits and related risk factors; appropriate ADR reporting and collaboration between allergists and pharmacovigilance centers can be a winning strategy for ADR prevention.

## 1. Introduction

Adverse drug reactions (ADRs) are defined as noxious and unintended responses to medicinal products [1], often causing hospitalization, extended hospital stays, additional clinical investigations, consultations, or treatments for related complications [2]. Therefore, they represent a significant public health concern with a considerable impact on healthcare systems both from clinical and economic perspectives [3]. Epidemiological data on the prevalence and incidence of ADRs vary globally, depending on the population groups, different types of drugs involved, the nature of the reactions, and the patients’ characteristics in terms of comorbidities or poly-drug treatment when experiencing an adverse drug reaction [4].

ADRs can be classified into two main categories: Type A, generally common and predictable adverse effects clearly related to the pharmacologic actions of the drug, and Type B, rare, around one third of ADRs, and usually unpredictable. The latter are mainly attributable to drug hypersensitivity reactions [4], not explained by the drug mechanism of action but involving the immune system with IgE or non-IgE-mediated immunological mechanisms [5]; they are relatively uncommon, but the prevalence in the community, and among different patients’ age groups, is unknown [6].

In principle, any pharmacological agent has the potential to elicit an adverse drug reaction. Nevertheless, drug classes most frequently associated with clinically significant adverse responses in allergy settings include mainly antimicrobials, anti-inflammatory drugs, antiepileptic agents, antineoplastic drugs, and, more recently, biological agents [7].

The World Allergy Organization (WAO) recommendations classify ADRs into immediate and delayed [8]. Immediate reactions occur within 1 h, though they can sometimes manifest within 6 h from drug exposure. Immediate reactions may be present phenotypically as urticaria, angioedema, bronchospasm, or, in serious cases, as anaphylaxis [9]. Delayed reactions typically occur several days or, sometimes, several weeks after drug exposure and usually are cutaneous, either mild, moderate, or severe [9]. Growing evidence on the increasing frequency of ADRs highlights the importance of awareness of their possible unpredictable occurrence, prevention, and appropriate management to ensure greater patient safety [10]. Several epidemiological studies have been published on this topic, even if these data are largely unverified due to variations in terminology and the frequent absence of clinical assessment or diagnostic testing [11]. Allergists in Drug Allergy Units usually investigate patients about previous ADRs, perform drug allergy testing, desensitization, and drug allergy de-labeling [12]. Reporting of ADRs is considered crucial for monitoring drug safety, contributing to pharmacovigilance activities, and detecting previously unknown ADRs [13]. In our Allergy and Clinical Immunology Unit, pharmacovigilance activities have always been carried out due to strict collaboration with the local Pharmacovigilance Center as part of the routine clinical practice. The present study was performed retrospectively to analyze spontaneous reports of ADRs, collected by interviewing patients referred to the Allergy and Clinical Immunology Unit of the “G. Martino” University Hospital of Messina, Italy, because of a positive history of ADR, from June 2022 to June 2024.

## 2. Materials and Methods

### 2.1. Study Design and Data Collection

A retrospective descriptive analysis of ADR reports from patients with a positive ADR history was carried out. All participants provided written informed consent before inclusion in the study; research objectives, privacy protections, and the use of anonymous data in compliance with the European General Data Protection Regulation (GDPR 2016/679), were approved by the Ethics Committee of the University Hospital of Messina (Protocol number 91/25).

The activity was coordinated by a multidisciplinary team of the University Hospital of Messina, including allergists, pharmacologists, and pharmacists, the latter also operating in the Sicily Regional Pharmacovigilance Center. Information collected from ADR reports included sex and age of the patients, any previous ADR or previously diagnosed allergies, presence of comorbidities, concomitant medications, suspected drugs, and clinical manifestations.

In accordance with the Italian pharmacovigilance legislation, all ADR reports were recorded into the national spontaneous reporting database (National Pharmacovigilance database) handled by the Italian Medicines Agency (AIFA), which ensures the collection, management, and analysis of spontaneous reports of suspected ADRs [14].

Drugs were codified by Anatomical-Therapeutic and Chemical classification (ATC) (level I–V) [15].

### 2.2. Adverse Drug Reactions Definitions and Outcome Measurement

Reported ADRs were classified using the terminology of the Medical Dictionary for Regulatory Activities (MedDRA) [16], evaluating both the System Organ Class (SOC) and Preferred Term (PT) levels. ADRs were considered serious if causing persistent/significant disability or incapacity, were life-threatening, or required inpatient hospitalization and prolongation of hospital stay, or any other important medical event, either based on clinical judgment or on MedDRA Important Medical Events (IME) list [17]. According to the time to onset (TTO), that is, the time from the start of drug administration to the beginning of any adverse event, ADRs were categorized as immediate, occurring within 6 h, or delayed, after 24 h. The Naranjo scale and the WHO causality assessment methodology for adverse events following immunization (AEFI) [18,19] were used to evaluate the causality relationship between each adverse event and implicated drug or vaccine administration, respectively. The Naranjo Scale uses a 10-question questionnaire to assign a score that categorizes causality as definite, probable, possible, or doubtful, while the WHO AEFI causality assessment algorithm helps to determine if the AEFIs have a consistent, inconsistent, or indeterminate causality relationship. Data collected from both tools were used to ensure the reliability and consistency of ADRs’ causality assessments.

### 2.3. Statistical Analyses

The Kolmogorov–Smirnov test was applied in order to check the normal distribution of the only numerical parameter, i.e., age; since it revealed a non-normal distribution, the non-parametric approach was adopted. Accordingly, the age was expressed as a median and interquartile range (Q1, Q3), and the categorical variables as absolute frequencies and percentages. The Chi-square test was used for statistical comparisons. An univariable and multivariable logistic regression model was estimated to identify possible significant predictors of serious ADR (yes or no). The following covariates were inserted in the univariate models: age, sex, cardiovascular diseases (yes or no), respiratory diseases (yes or no), cancer (yes or no), autoimmune diseases (yes or no), previous allergies (yes or no), previous ADRs (yes or no), number of concomitant diseases, number of concomitant drugs. All the explanatory variables tested in the univariate approach were included in the multivariable model in order to identify independent significant predictors of ADR seriousness. The results of univariable and multivariable logistic regression models were expressed as odds ratios (ORs), 95% confidence intervals (CIs) for OR, and significance. A *p*-value lower than 0.050 was considered statistically significant. Statistical analyses were performed using SPSS for Window, version 22.

## 3. Results

A total of 388 spontaneous ADR reports, mostly from females (276; 71.1%), were collected. In all, patients reporting ADRs had a median age of 45 years [IQR: 29.8–57.0]. ADR reports were mostly from 18 to 64-year-old adult patients (327; 84.3%), followed by elderly patients aged ≥65 years (47; 12.1%), and adolescents <18 years (14; 3.6%). Main comorbidities included cardiovascular diseases in 113 (29.1%) reports, autoimmune diseases in 72 (18.6%), dyslipidemia in 58 (14.9%), respiratory diseases in 36 (9.3%), diabetes in 31 (8.0%), and cancer in 23 (5.9%); 127 (32.7%) reports did not describe any comorbidity.

Most frequent concomitant medications to manage comorbidities included Renin-Angiotensin System drugs in 58 ADR reports (14.9%), Lipid-modifying agents in 39 (10.1%), Drugs for acid-related disorders in 37 (9.5%), antidiabetics in 33 (8.5%), Thyroid therapy in 33 (8.5%), and antithrombotics in 30 (7.7%).

A positive history of allergies other than ADRs (78.6%) and/or a past medical history of ADRs (68%) were also present (Table 1).

Serious ADR reports were 149 (38,4%); 19/149 (12.7%) had caused hospitalization or prolonged it, 126/149 (84.6%) were ADR with clinical pictures from the MedDRA IME list, and 4/149 (2.7%) had been life-threatening. Outcome of serious ADRs was a complete resolution in 379 (97.7%) reports, improvement in 3 (0.8%), not yet recovered/persisting in 5 (1.3%), without any significant difference between serious and non-serious ADRs; in 1 report, the outcome was missing. ADR causality using the Naranjo algorithm for drugs resulted in probable in 211 reports (54.4%), possible in 166 (42.8%), and definite in 7 (1.8%), while vaccine causality using the WHO AEFI causality assessment algorithm resulted in a consistent causal association to immunization in 2 reports and indeterminate in 2 others (Table 2).

Overall, the 388 ADR reports described reactions to 439 suspected drugs. Most ADR reports, 353 out of 388, included a single suspected drug, 25, two drugs, 5, three, while the other 5 reports listed four or more suspected drugs, 4 reports were associated with vaccines.

The culprit drugs reported were classified according to ATC 2nd level. Antibacterials for systemic use, including mainly beta-lactams (71.1%), macrolides (17.8%), quinolones (8.1%), and anti-inflammatory drugs, were both indicated with the same frequency (30.8% each), followed by analgesics (8.4%) and anesthetic drugs (5.9%). No significant differences for ADR seriousness were identified by each therapeutic group (Table 3). The most reported drugs, calculated on the total of all reports, were amoxicillin/clavulanic acid (12.1%), ibuprofen (9.6%), ketoprofen (8.9%), diclofenac (6.4%), paracetamol (4.3%), clarithromycin (3.9%), and ceftriaxone and amoxicillin (3%).

**Figure 1:**
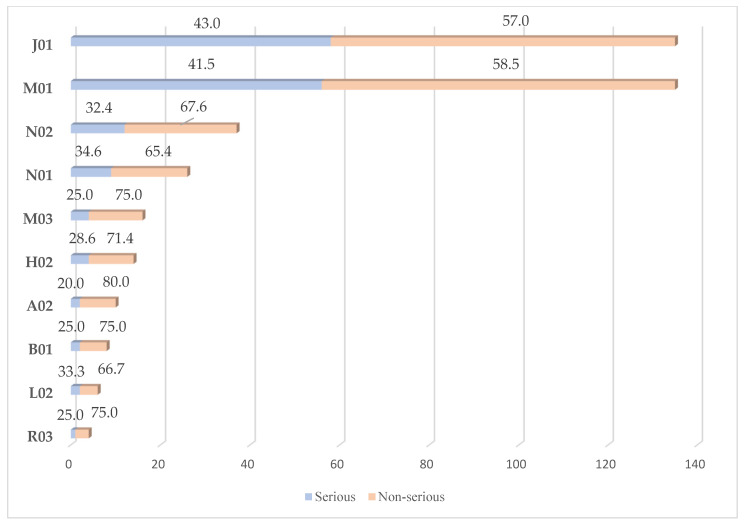
Proportions of serious ADR reports within each therapeutic group (ATC 2nd level). Legend. J01—Antibacterials for systemic use; M01—Anti-inflammatory and antirheumatic drugs; N02—analgesics; N01—anesthetics; M03—Muscle relaxants; H02—Corticosteroids for systemic use; A02- Drugs for acid related disorders; B01—Antithrombotics; L02—Endocrine therapy; R03—Drugs for obstructive airways disorders.

Considering serious ADR reports within each therapeutic group, antibacterial drugs included the largest within-class proportion of serious cases (43%), followed by anti-inflammatory drugs (41.5%), anesthetics (34.6%), and analgesics (32.4%) (Figure 1).

**Figure 2:**
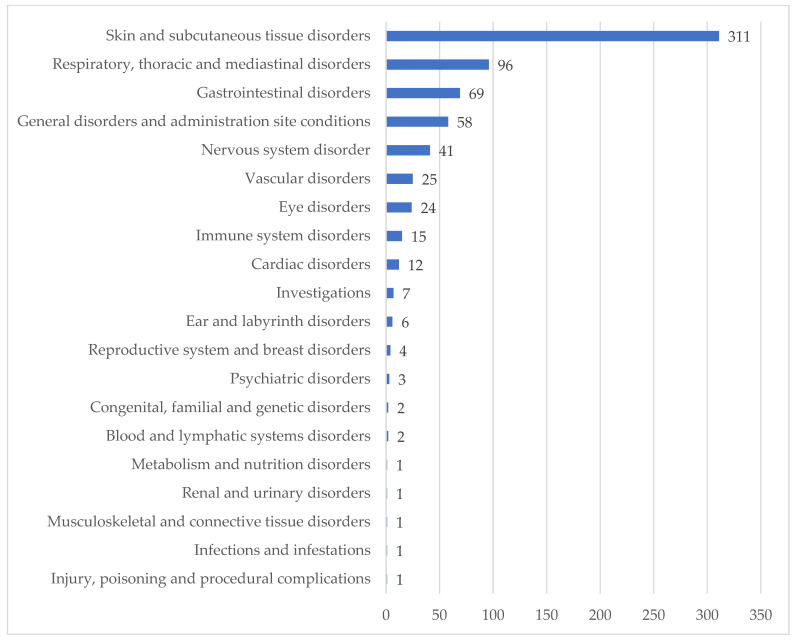
ADRs classified by System Organ Class (SOC) ^a^. ^a^ The total number of ADRs reported in the figure does not coincide with the total number of reports because in some individual reporting forms, more than one ADR was indicated.

ADR reactions according to the clinical manifestations were classified using the MEDRA SOC level. Reports described mostly ADR with skin and subcutaneous tissue manifestations (311; 45.7%), followed by respiratory (96; 14.1%), gastrointestinal (69; 10.2%), and general disorders (58; 8.5%) (Figure 2).

**Figure 3:**
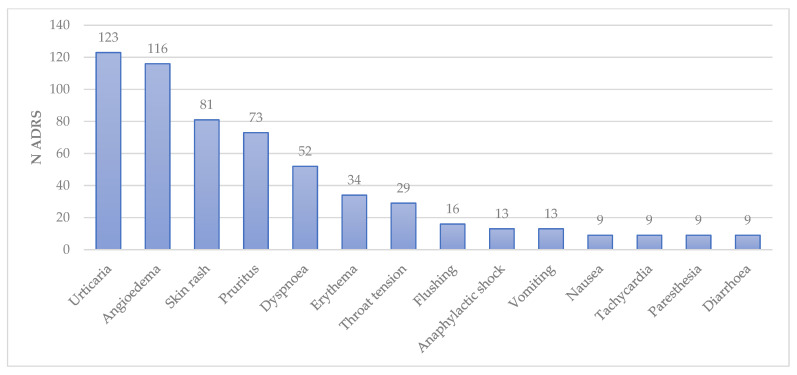
Most common preferred terms (N ADR ≥ 9) in ADR reports.

The most common terms used to describe ADRs according to the MEDRA PT level were urticaria (123; 31.7%), angioedema (116; 29.9%), skin rash (81; 20.9%), pruritus (73; 18.8%), dyspnea (52; 13.4%), erythema (34; 8.8%), throat tension (29; 7.5%), flushing (16; 4.1%), anaphylactic shock (13; 3.4%), vomiting (13: 3.4%), nausea (9; 2.3%), tachycardia (9; 2.3%), paresthesia (9: 2.3%), diarrhea (9; 2.3%) (Figure 3).

The TTO of ADRs described in reports was assessed for each drug therapeutic group; 413 (71.3%) ADR were classified as immediate and 166 (28.7%) as delayed. Reports of immediate reactions were mostly correlated to antibacterial for systemic use and anti-inflammatory drugs, while delayed reactions were correlated to endocrine therapy, vaccines, and antiepileptics (Supplementary Material, Table S1).

Among immediate reactions, anaphylactic shock was present in 13 ADR reports (3.35%), 3 from amoxicillin-clavulanate acid, 3 from ceftriaxone, and the others from erythropoietin, sodium metamizole, piperacillin-tazobactam, rocuronium bromide, sodium diclofenac, doxycycline, and Hymenoptera venom immunotherapy extract.

To identify significant predictors of serious ADR, a univariate and multivariable logistic regression model was applied, including covariates such as age, sex, the presence of cardiovascular, respiratory, cancer, or autoimmune diseases, a positive history of allergy and previous ADRs, number of concomitant diseases, and number of concomitant drugs. A statistically significant result was obtained. In the final multivariable model, a higher risk of developing serious ADRs was found in elderly patients aged ≥65-years (OR = 1.017; *p* = 0.012); specifically, these elderly patients affected by serious ADRs all had comorbidities, and as clinical manifestations of ADRs, mainly angioedema and dyspnea, while in two cases, anaphylactic shock and in one case respiratory failure, while a positive history of allergy (OR = 0.563; *p* = 0.023) and past medical history of ADRs (OR = 0.504; *p* = 0.045) were negatively correlated to the occurrence of a serious ADR (Table 4).

## 4. Discussion

No drug is entirely without risk; even when used appropriately, it can potentially cause an ADR [20]. In the literature, there are not many pharmacovigilance studies carried out in collaboration between allergists and pharmacologists, but spontaneous reporting should be encouraged [21]. In allergy settings, a comprehensive assessment of patients with a history of suspected ADRs is usually carried out; patients’ characteristics and the medications involved in the ADRs are therefore obtained [22]. The observational retrospective study described in this manuscript concerns ADRs collected by allergists, as part of the anamnestic interview of patients examined at the Drug Allergy Clinic of the Allergy and Clinical Immunology Unit of the G. Martino University Hospital of Messina, Italy. ADR were spontaneously reported to pharmacists, from the Pharmacovigilance Center of the same hospital, to record data in the Italian pharmacovigilance database.

It was observed that most ADR reports were from adult patients, and this may explain why most reports regarded patients with at least one or more comorbidity. Osanlou R. et al. [23] described polypharmacy as one of the causes of ADR; in our population, this data was not confirmed, as most ADR reports, 353 out of 388, were correlated to a single suspected drug.

ADRs reports in our study were also mostly from female patients. This last result corresponds to the already published literature also available from the Eudravigilance database, which shows a higher prevalence of ADRs in females compared to males [24,25]. Sex-related factors such as hormones, genetics, metabolic processes, anatomical characteristics, and organ function influence pharmacokinetic processes, and all these factors may account for the differing likelihood of experiencing ADRs between females and males [26]. Additionally, physicians’ awareness of the implications of sex differences on dosing and adverse event monitoring in routine practice needs improvement; therefore, pharmacovigilance studies can give important information [27].

Antibiotics and anti-inflammatory drugs were the most reported drugs causing ADR, in line with other surveillance data [28], usually, anti-inflammatory drugs are, after antibiotics, the second cause of hypersensitivity reactions in the general population [29]. The most common drugs involved in ADRs were amoxicillin/clavulanic acid, ibuprofen, ketoprofen, diclofenac, paracetamol, clarithromycin, ceftriaxone, and amoxicillin; these drug molecules are also reported as frequently causing ADR in other Italian post-marketing studies [9,30].

Regarding antibiotics for systemic use, these drugs are used in all age groups and represent medications to which people may be frequently exposed [31]. This data could also be correlated to a higher consumption of antibiotics, around 10% more, in Italy than in other European countries, according to the ESAC-NET annual epidemiological report for 2024 [32]. In our analysis, ADRs from beta-lactams such as penicillins and cephalosporins were the most frequently reported.

The most common clinical manifestations induced by antibiotics for systemic use and anti-inflammatory drugs were urticaria and angioedema, with skin and subcutaneous tissue involvement, respectively [33]; in general, dermatologic manifestations are the most common presentations of ADRs [34].

Anaphylactic shock was mainly associated with penicillin and cephalosporins; this data is in line with the literature data [35]. In one case, anaphylactic shock was a consequence of Hymenoptera Venom Immunotherapy treatment [36], a rare cause of anaphylaxis, mainly correlated to the patient’s elevated basal serum tryptase levels [37]. ADR anaphylaxis is an important issue and should be prevented, also to avoid patients’ psycho-cognitive involvement and quality of life impairment [38].

Older age seems to be associated with a significantly increased risk of hospitalization [39]; a study carried out in the regional Center of Pharmacovigilance of Bordeaux demonstrated that age of 75 years and more was a risk factor of serious ADR occurrence [40], probably due to comorbidities and the use of multiple medications in elderly patients [41].

The results of the multivariate logistic regression model, applied to ADR data from the present study, confirm that older age was the unique predictive factor of a serious adverse event. Conversely, a positive history for allergies other than ADR and past ADR history were negatively correlated with successive serious ADR. The latter seems to be an original result, not yet confirmed, but in our opinion, it can be considered relevant. It is likely that if patients have already been diagnosed as allergic or have had a previous ADR, they are better informed about the possibility of ADRs and are more cautious when taking a medicine drug [42]. It is reported that 10% of the general population has a positive history of ADR, but unfortunately, only a few drug allergy units exist where patients can be correctly diagnosed with drug allergies [43]. Drug allergy settings are also of great importance for eventually re-testing patients with suspected drugs, offering possible de-labeling for previous unclear drug reactions [44].

Our findings highlight the need for healthcare systems to develop and implement structured approaches for monitoring drug-induced hypersensitivity reactions, particularly those related to beta-lactams, NSAIDs, analgesics, and other medications frequently associated with allergic responses.

### Strengths and Limitations

This study adds important information to the general knowledge about the impact of ADRs in an allergy setting. Adopting an active ADR surveillance approach, we provided additional evidence concerning several drug safety issues.

A multidisciplinary team—comprising pharmacists and clinical pharmacologists from a Pharmacovigilance Center, together with allergists—contributed to the study. This collaboration facilitated the collection of detailed medication histories, the monitoring of polypharmacy, and the acquisition of additional data essential for accurate ADR characterization and drug–ADR causality assessment. The integration of allergists’ clinical evaluations with the expertise of pharmacovigilance specialists likely strengthened the overall clinical interpretation.

Such an approach may also help mitigate well-known limitations of passive ADR surveillance systems, including underreporting, insufficient quality and completeness of reports, and the lack of confirmed ADR diagnoses.

However, some limitations should be acknowledged. This is a retrospective, single-center study with a homogeneous assessment and no comparative control group. These factors restrict the generalizability of the findings, as the included patients may not fully represent the broader population. Nonetheless, our results are consistent with those reported in other international studies.

The number of predictors analyzed for serious ADR was also limited, and additional variables may influence the occurrence of serious adverse events. Patterns of drug consumption in the general population could have contributed to the observed ADR frequencies; for instance, the widespread self-medication with anti-inflammatory drugs and the overuse of antibiotics in Italy may have increased the frequency of ADRs associated with these drug classes.

## 5. Conclusions

Information about pharmacovigilance activities is useful for raising awareness among both health professionals and the general population on the importance of reporting ADRs. The role of allergologists in pharmacovigilance activities is relevant and contributes to monitoring the safety profile of drugs in clinical practice. In our study, the prevalence of drug allergy resulted in higher rates in female patients and adults, with most cases attributed to antibiotics and anti-inflammatory drugs. Cutaneous signs and symptoms resulted as the most common manifestations of ADRs, a few cases of anaphylaxis were registered, and respiratory events.

## Figures and Tables

**Table 1:** Patients’ characteristics in ADR reports included in the study (total number of reports: 388).

Characteristics		Value n	Frequency %
Sex	Female	276	71.1
	Male	112	28.9
Age groups (years)	16–18	14	3.6
	18–64	327	84.3
	≥65	47	12.1
Main comorbidities *	Cardiovascular diseases	113	29.1
	Autoimmune diseases	72	18.6
	Dyslipidemia	58	14.9
	Respiratory diseases	36	9.3
	Diabetes	31	8.0
	Cancer	23	5.9
Main concomitant medications **	Renin–Angiotensin System drugs	58	14.9
	Lipid-modifying substances	39	10.1
	Drugs for acid-related disorders	37	9.5
	Antidiabetics	33	8.5
	Thyroid therapy	33	8.5
	Antithrombotics	30	7.7
Positive history of allergies	Yes	305	78.6
	No	83	21.4
Past medical history of ADRs	Yes	264	68.0
	No	124	32.0

* Most frequent comorbidities are indicated; one patient could be affected by more than one concomitant disease. ** Most frequent concomitant medications are indicated; one patient could use more than one concomitant medication.

**Table 2:** Sex and age distribution, causality, and outcomes in reports of serious ADRs.

	Total N 388 (%)	Serious N 149 (%)	Non-Serious N 239 (%)	*p* Value
Sex				
Female	276 (71.1)	99 (66.4)	177 (74.1)	0.107
Male	112 (28.9)	50 (33.6)	62 (25.9)	
Age groups (years)				
<18	14 (3.6)	7 (4.7)	7 (2.9)	0.363
18–64	327 (84.3)	118 (79.2)	209 (87.5)	0.030
≥65	47 (12.1)	24 (16.1)	23 (9.6)	0.057
Outcomes of ADRs				
Complete resolution	379 (97.7)	147 (98.6)	232 (97.1)	0.312
Improvement	3 (0.8)	1 (0.7)	2 (0.8)	0.856
Not yet recovered/persisting	5 (1.3)	1 (0.7)	4 (1.7)	0.394
Not available (N.A.)	1 (0.2)	-	1 (0.4)	N.A.
ADR Causality				
**Drugs**				
Possible	166 (42.8)	66 (44.3)	100 (41.8%)	0.672
Probable	211 (54.4)	78 (52.3)	133 (55.6)	0.526
Certain	7 (1.8)	3 (2.0)	4 (1.7)	0.060
**Vaccines**				
Consistent	2 (0.5)	1 (0.7)	1 (0.4)	0.735
Indeterminate	2 (0.5)	1 (0.7)	1 (0.4)	0.735

**Table 3:** Therapeutic groups (classified according to ATC 2nd level) are associated with adverse drug reactions and stratified by ADR seriousness.

Therapeutic Groups (ATC Level 2)	Total Reports ^a^ N 388 (%)	Serious ADR Reports N 149 (%)	Non-Serious ADR Reports N 239 (%)	*p*-Value ^b^
Antibacterials for systemic use	135 (30.8)	58 (34.3)	77 (28.5)	0.215
Antinflammatory and antirheumatic drugs	135 (30.8)	56 (33.2)	79 (29.3)	0.061
Analgesics	37 (8.4)	12 (7.1)	25 (9.3)	0.544
Anesthetics	26 (5.9)	9 (5.3)	17 (6.3)	0.840
Muscle relaxants	16 (3.6)	4 (2.4)	12 (4.4)	0.388
Corticosteroids for systemic use	14 (3.2)	4 (2.4)	10 (3.7)	0.624
Medications for acidity-related disorders	10 (2.3)	2 (1.2)	8 (3.0)	0.377
Antithrombotics	8 (1.8)	2 (1.2)	6 (2.2)	0.674
Endocrine therapy	6 (1.4)	2 (1.2)	4 (1.5)	0.999
Drugs for obstructive airways disorders	4 (0.9)	1 (0.6)	3 (1.1)	0.970
Other drugs for the nervous system	4 (0.9)	2 (1.2)	2 (0.7)	0.999
Vaccines	4 (0.9)	2 (1.2)	2 (0.7)	0.999
Stomatological preparations	4 (0.9)	2 (1.2)	2 (0.7)	0.999
Psycholeptics	3 (0.7)	2 (1.2)	1 (0.4)	0.678
Contrast media	3 (0.7)	-	3 (1.1)	n.a.
Allergens	3 (0.7)	1 (0.6)	2 (0.7)	0.999
Agents acting on the renin-angiotensin system	3 (0.7)	1 (0.6)	2 (0.7)	0.999
Antimycobacterials	2 (0.5)	-	2 (0.7)	n.a.
Blood substitutes and perfusion solutions	2 (0.5)	1 (0.6)	1 (0.4)	0.999
Antiepileptics	2 (0.5)	1 (0.6)	1 (0.4)	0.999
Anti-anemic drugs	2 (0.5)	1 (0.6)	1 (0.4)	0.999
Cardiac therapy	2 (0.5)	-	2 (0.7)	n.a.
Drugs for the treatment of bone diseases	2 (0.5)	1 (0.6)	1 (0.4)	0.999
Cough and cold preparations	1 (0.2)	-	1 (0.4)	n.a.
Other dermatological preparations	1 (0.2)	-	1 (0.4)	n.a.
Antigoutosis	1 (0.2)	-	1 (0.4)	n.a.
Antiprurigins, including antihistamines, anesthetics, etc.	1 (0.2)	1 (0.6)	-	n.a.
Antifungals for dermatological use	1 (0.2)	-	1 (0.4)	n.a.
Antifungals for systemic use	1 (0.2)	-	1 (0.4)	n.a.
Ophthalmological	1 (0.2)	1 (0.6)	-	n.a.
Immunosuppressors	1 (0.2)	1 (0.6)	-	n.a.
All other therapeutic products	1 (0.2)	-	1 (0.4)	n.a.
Sex hormones and modulators of the genital system	1 (0.2)	1 (0.6)	-	n.a.
Throat preparations	1 (0.2)	1 (0.6)	-	n.a.
Urological drugs	1 (0.2)	-	1 (0.4)	n.a.
**Total drugs**	**439 (100)**	**169 (100)**	**270 (100)**	

Legend: n.a.: not applicable. ^a^ The total number of drugs reported in Table 3 is higher than the total number of reports, as in the reporting form, more than one drug was sometimes indicated as the suspected cause of ADRs. ^b^ *p*-value: calculated by chi-square test with Yates correction: level of significance < 0.005.

**Table 4:** Results of uni- and multi- variable logistic regression model for identification of significant predictors of serious ADRs.

	Univariable			Multivariable		
Variables	OR	95% CI	*p*-Value	OR	95% CI	*p*-Value
Age	1.016	1.003–1.029	0.013	1.017	1.004–1.030	0.012
Sex	0.694	0.444–1.084	0.108	0.780	0.484–1.259	0.309
Cardiovascular diseases	1.491	0.617–3.602	0.375	1.350	0.476–3.826	0.573
Respiratory diseases	0.692	0.348–1.378	0.295	0.713	0.344–1.480	0.364
Cancer	0.986	0.399–2.439	0.976	0.973	0.345–2.744	0.958
Autoimmune diseases	0.647	0.334–1.255	0.198	0.649	0.318–1.326	0.235
Previous allergies	0.562	0.344–0.917	0.021	0.563	0.344–0.924	0.023
Past medical history of ADRs	0.516	0.334–0.798	0.003	0.504	0.257–0.985	0.045
Number of concomitant diseases	0.966	0.865–1.079	0.540	0.939	0.805–1.096	0.425
Number of concomitant drugs	1.030	0.921–1.152	0.606	1.001	0.862–1.162	0.987

## Data Availability

Data available after reasonable request.

## Supplementary Materials

The following supporting information can be downloaded at: https://www.mdpi.com/article/10.3390/jcm15020848/s1, Table S1: Time to onset of all adverse drug reactions for each therapeutic group (ATC 2nd Level).
