# Supplementary material for: A Two-Year Pharmacovigilance Analysis of Adverse Drug Reactions Reported from a University Allergy Setting
Source: J Clin Med. 2026 Jan 20;15(2):848. doi: 10.3390/jcm15020848 (PMC12842238; doi:10.3390/jcm15020848)
Supplement: Supplementary file 1 [file jcm-15-00848-s001.zip › jcm-4089010-supplementary.pdf]

Table S1. TTO of all ADRs for each therapeutic group (ATC 2nd Level) [a].

ATC Level 2	Therapeutic class	Immediate [b] N 413 (%)	Delayed [c] N 166(%)
**J01**	Antibacterials for systemic use	155 (46.27)	59 (17.61)
**M01**	Antinflammatory and antirheumatic drugs	115 (39.93)	37 (12.85)
**N02**	Analgesics	19 (28.36)	2 (2.98)
**N01**	Anaesthetics	35 (72.92)	3 (6.25)
**M03**	Muscle relaxants	24 (64.86)	-
**A02**	Drugs for acid related disorders	16 (47.06)	-
**H02**	Corticosteroids for systemic use	6 (23.08)	9 (34.61)
**L02**	Endocrine therapy	4 (25.00)	11 (68.75)
**J07**	Vaccines	2 (18.18)	9 (81.82)
**B01**	Antithrombotics	3 (27.28)	4 (36.36)
**V01**	Allergens	7 (70.00)	-
**V08**	Contrast media	7 (70.00)	-
**R03**	Drugs for obstructive airways disorders	3 (30.00)	5 (50.00)
**C09**	Agents acting on the renin-angiotensin system	-	-
**N07**	Other drugs for the nervous system	-	4 (50.00)
**N03**	Antiepileptics	-	7 (100)
**B05**	Blood substitutes and perfusion solutions	-	-
**M05**	Drugs for the treatment of bone diseases	2 (40.00)	3 (60.00)
**J04**	Antimycobacterials	-	2 (40.00)
**C01**	Cardiac therapy	-	-
**N05**	Psycholeptics	3 (75.00)	-
**R02**	Throat preparations	-	4 (100)
**A01**	Stomatological preparations	1 (25.00)	-
**D11**	Other dermatological preparations	3 (100)	-
**L04**	Immunosuppressors	-	3 (100)
**D04**	Antipruritics, including antihistamines, anesthetics, etc.	3 (100)	-
**M04**	Antigout preparations	-	3 (100)
**S01**	Ophthalmologicals	-	-
**J02**	Antifungals for systemic use	2 (100)	-
**D01**	Antifungals for dermatological use	2 (100)	-
**B03**	Anti-anaemic drugs	1 (50.00)	1 (50.00)
**G04**	Urological drugs	-	-
**V03**	All other therapeutic products	-	-
**G03**	Sex hormones and modulators of the genital system	-	-

[a] Out of 388 reports, only 203 have an assessable TTO, containing 237 drugs and 514 ADRs, resulting in 579 drug–ADR pairs. [b] Immediate reactions: early ADR onset within 24 h after drug exposure. [c] Delayed reactions: late ADR onset after 24 h after drug exposure.
